# A Mobile App for Assisting Users to Make Informed Selections in Security Settings for Protecting Personal Health Data: Development and Feasibility Study

**DOI:** 10.2196/11210

**Published:** 2018-12-11

**Authors:** Leming Zhou, Bambang Parmanto, Zakiy Alfikri, Jie Bao

**Affiliations:** 1 Department of Health Information Management University of Pittsburgh Pittsburgh, PA United States

**Keywords:** data security, mobile app, education, feasibility studies

## Abstract

**Background:**

On many websites and mobile apps for personal health data collection and management, there are security features and privacy policies available for users. Users sometimes are given an opportunity to make selections in a security setting page; however, it is challenging to make informed selections in these settings for users who do not have much education in information security as they may not precisely know the meaning of certain terms mentioned in the privacy policy or understand the consequences of their selections in the security and privacy settings.

**Objective:**

The aim of this study was to demonstrate several commonly used security features such as encryption, user authentication, and access control in a mobile app and to determine whether this brief security education is effective in encouraging users to choose stronger security measures to protect their personal health data.

**Methods:**

A mobile app named *SecSim* (Security Simulator) was created to demonstrate the consequences of choosing different options in security settings. A group of study participants was recruited to conduct the study. These participants were asked to make selections in the security settings before and after they viewed the consequences of security features. At the end of the study, a brief interview was conducted to determine the reason for their selections in the security settings. Their selections before and after the security education were compared in order to determine the effectiveness of the security education. The usability of the app was also evaluated.

**Results:**

In total, 66 participants finished the study and provided their answers in the app and during a brief interview. The comparison between the pre- and postsecurity education selection in security settings indicated that 21% (14/66) to 32% (21/66) participants chose a stronger security measure in text encryption, access control, and image encryption; 0% (0/66) to 2% (1/66) participants chose a weaker measure in these 3 security features; and the remainder kept their original selections. Several demographic characteristics such as marital status, years of experience using mobile devices, income, employment, and health status showed an impact on the setting changes. The usability of the app was good.

**Conclusions:**

The study results indicate that a significant percentage of users (21%-32%) need guidance to make informed selection in security settings. If websites and mobile apps can provide embedded security education for users to understand the consequences of their security feature selection and the meaning of commonly used security features, it may help users to make the best choices in terms of security settings. Our mobile app, SecSim, offers a unique approach for mobile app users to understand commonly used security features. This app may be incorporated into other apps or be used before users make selections in their security settings.

## Introduction

### Background

In recent years, health data breaches have begun to occur more frequently, impacting a growing number of people. From February 10, 2016, to February 6, 2018, there were 2201 reported Protected Health Information (PHI) breaches in the United States and each affected 500 or more individuals; in total, more than 177 million Americans (54.1% of the US population) across the nation were affected by these PHI breaches [1].

PHI breaches are costly to industries. For example, according to the 2017 Cost of Data Breach Study released by IBM Security and the Ponemon Institute, the average global cost of a health data breach per lost or stolen record was US $380 [2]. Overall, the US health care industry spent approximately US $67 billion dealing with issues triggered by PHI breaches on activities such as conducting investigations, notifying customers, recovering data, subscribing to credit monitoring services for customers, hiring knowledgeable security personnel, and strengthening the security measures of information technology (IT) systems.

There are many good approaches to reduce the number of PHI breaches such as using a highly qualified security incident response team, extensively using encryption in the IT system, and providing security training to health IT system users [2]. Compared with the first approach (using highly qualified security experts), providing security training to health IT system users tends to be a very cost-efficient and effective approach. After all, among these PHI breaches in the last 2 years, only 19% were because of hacking or IT incidents, which are handled by security incident response teams. The other, more than 80%, were because of issues on the user end such as improper disposal of PHI, theft or loss of devices, and incidents of unauthorized access or disclosure [1].

The security education of users is particularly important as smartphones and tablets are widely used in the health care industry for PHI access. By the end of 2017, 77% of Americans owned a smartphone and 53% of Americans owned a tablet computer, compared with those in 2011, when ownership of these 2 mobile computing devices was just 35% and 8%, respectively [3]. As the mobile user population has grown, smartphones and tablets have become popular within the health care domain for both providers and patients. According to a recent survey study of 3800 physicians, 83% owned at least one mobile device and 25% of these physicians used both smartphones and tablets within their clinical practice [4]. Similarly, many patients use their mobile devices to receive health care services [5]. As the health care–related uses increase and more sensitive information is accessed via mobile devices, there is a growing need for users (both health care providers and patients) to be conscious of information security.

Previous studies have indicated that mobile health (mHealth) app users, especially patients, are concerned about their health data security and their individual privacy, and some users choose not to use mHealth apps because of this concern [5-8]. mHealth app users’ perception of security and privacy are highly contextual and are related to multiple demographics such as age, gender, income, race, health status, and education [9-11].

On the technical end, mHealth apps and mobile operating systems offer various security features such as passcodes, usernames and passwords, data encryption, and remote wiping. Researchers have also provided detailed security recommendations for mHealth app development in particular [12]. However, many smart device users did not use even the most basic authentication features (such as a passcode) to prevent the access of private data on their mobile devices [13-15]. In other words, security features are available to mHealth app users, but the problem is whether these users are capable of using these security features to protect the PHI.

On today’s websites and mobile apps, a security setting page and privacy policy are often provided to users. Examples of the security setting page are the “Touch ID & Passcode” page in iOS and the “Sign-in & Security” page in Gmail.

Privacy policies detail a website’s or mobile app’s specific practices with regards to data collection, storage, and use. It is assumed that users of the website or mobile app would be able to understand the content of these privacy policies. These privacy policies can be very useful for people who can understand the security terms and technologies such as encryption, access control, and security protocol names. However, for people who have not had a chance to receive formal education in information security (a majority of people), it is fairly challenging to fully understand the content of the privacy policy, let alone make an informed selection in the security settings. A specific example from Apple demonstrates this. In Apple’s privacy policy updated on May 22, 2018, it stated that “Apple online services such as the Apple Online Store and iTunes Store protect your personal information during transit using encryption such as Transport Layer Security (TLS). When your personal data is stored by Apple, we use computer systems with limited access housed in facilities using physical security measures. With the exception of iCloud Mail, iCloud data is stored in encrypted form including when we utilize third-party storage.” [16] Even if ignoring the specific name of the protocol (eg, TLS), most people will still wonder what the terms *encryption*, *encryption during transit*, and *limited access* mean.

The other assumption on privacy policy is that users will read the privacy policy carefully and make the proper selections for their PHI on the security setting page according to their security and privacy needs. However, an earlier study also indicated that participants often install mobile apps from unfamiliar vendors without reading the app’s privacy policies [17]. The existence of such a high number of PHI breaches also indicates that this assumption is not true.

People can make proper selection in security settings only if they have sufficient knowledge on this topic. However, most people do not receive formal, intensive security education. Even health care providers, who typically receive training on the Health Insurance Portability and Accountability Act regulations and the specific policies of their organization, do not receive much training on information security itself. In other words, many people do have the general idea that there is a potential for security risks. If they do not have a clear idea about how to use the protection provided by specific security features, they may take risky actions or intentionally sacrifice their information security and privacy for convenience or a small amount of financial benefits [18].

### Objectives

The purpose of this study was to determine whether a brief security education offered in a mHealth app can change users’ behavior in choosing security settings to improve the current situation. Our hypothesis is that mHealth app users can benefit from this brief and informal security education, and once they receive such education, many of them will choose a stronger security measure if they did not do so initially. Here *users* can be both health care providers and patients.

## Methods

### Features of the Mobile App

In this study, we chose a few commonly used security features in health IT systems and implemented a simulation or demonstration of these features in a mobile app named SecSim (Security Simulator). The chosen security features were as follows: (1) data encryption (a process of converting plain text into something that appears to be random and meaningless), (2) user authentication (a process that allows an entity, such as a Web server, to verify the identity of someone), (3) access control (the selective restriction of access to information or other resources), and (4) image encryption (a process of hiding the meaning of a private or sensitive image). We also elicited study participants’ opinions on password update frequency and preferred data storage and backup locations.

This mobile app, SecSim, has several major components, including (1) pages for registering the users and collecting the individual’s personal health data, which is the information users wants to protect in the app; (2) pages for simulating or demonstrating the chosen security features; (3) pages offering the user security setting options; (4) a log-in page; and (5) a page with a summary of security settings chosen. The order of running these components was 1-4-3-2-3-5. In other words, first the user registers a unique account and enters some personal health information. Then, the user can log in and make selections in security settings, the same as he or she does when using other mobile apps or websites. These actions take place before the security education (or where the education mode is not activated). In the next step, the user goes through each of the implemented security features and views the consequences of their selection. This is the mode where the security education takes place (or where the education mode is activated). At the end of the demonstration, the user is given a chance to make another round of selections in the security settings and view the summary of his or her selections.

**Figure 1:**
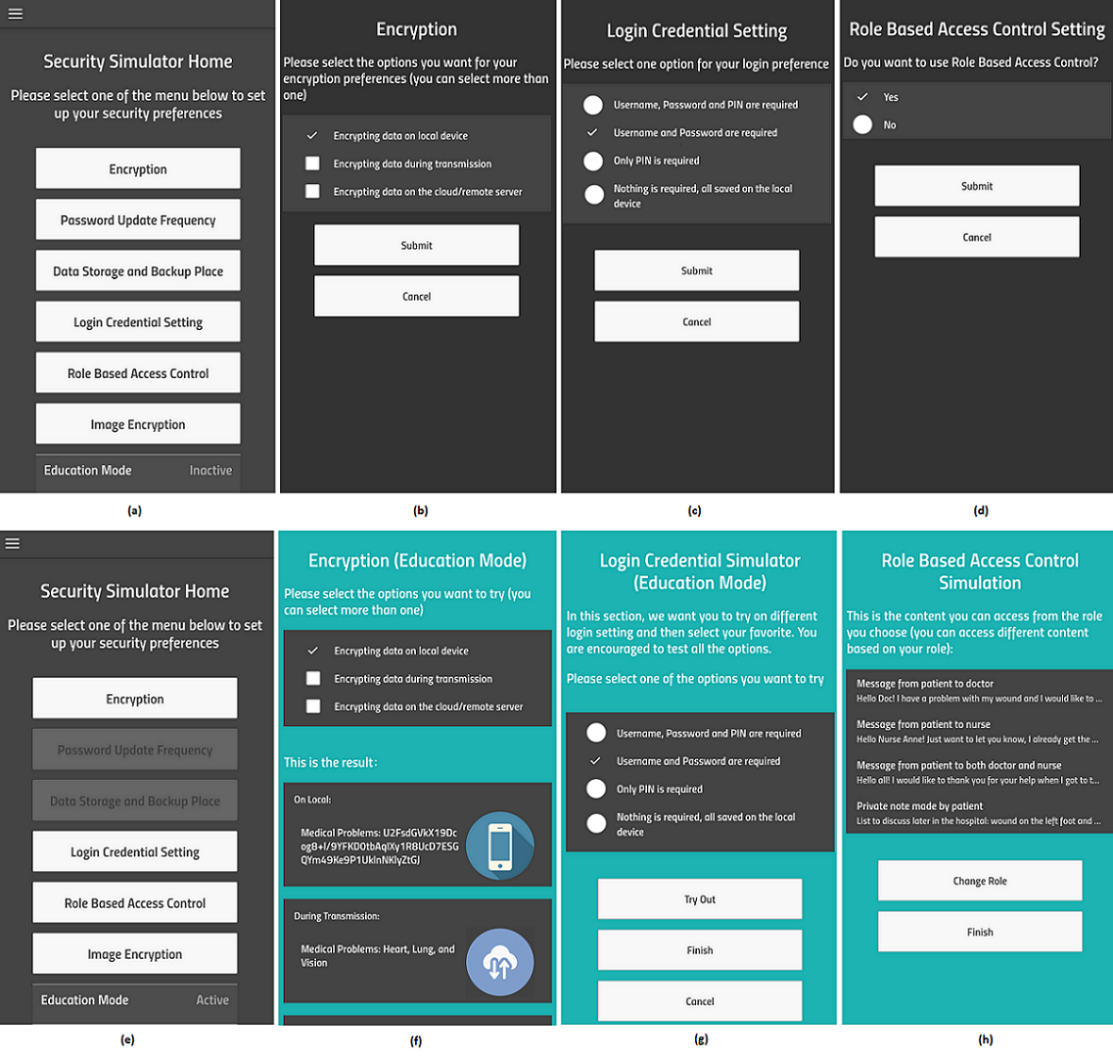
Screenshots for the list of security features and the options and simulation of 3 security features.

Figure 1 shows some screenshots of the app’s components. The top 4 screenshots show options available in (a) the main page of the app, (b) the encryption page, (c) the log-in credentials page, and (d) the role-based access control (RBAC) page when the education model is not activated. In this mode, users simply make their selections according to their own understanding. Once the security education mode is activated, the corresponding pages are updated. The bottom 4 screenshots show the following updated contents: (e) the options available on the main page, (f) the simulation for when different encryption options are selected, (g) the simulation for when different combinations of log-in credential are selected, and (h) a page of the RBAC simulation. On the encryption page, when no option is selected, all the contents are shown in clear text (not shown). If one of the options in (f) is selected, the contents at this location are shown as cipher texts and others are still shown as clear text (the content on the remote server is at the lower part of the screen, not shown in the screenshot). On the log-in credential simulator page (g), after one option is selected, the corresponding log-in page is shown, and the user is required to enter the log-in credentials accordingly to enter the system. On the RBAC simulation page (h), the user can choose one of the 3 roles: patient, physician, or nurse, and the corresponding content is shown in a new page (h). Not included in the figure are the pages for log-in, user registration or data collection, image encryption, the details of the log-in simulation and RBAC simulation, and the security settings summary page. Below is a detailed description of the options available corresponding to the implemented security features in the SecSim app.

First, there are 5 options for password update frequency: (1) once a month, (2) once every 3 months, (3) once every 6 months, (4) once a year, and (5) never. There are 3 options for data storage and backup location: (1) on local drive only, (2) on remote server only, and (3) both on local drive and remote drive.

On the encryption page, there are 3 options for data encryption: (1) applying encryption on local device (mobile device), (2) applying encryption during the transmission, and (3) applying encryption on the cloud server. Users could choose none, one, or more than one of these 3 options. For this specific question, the more options selected, the stronger the security. In other words, encrypting data at all three places is the strongest, encryption data at any two places is not as strong, and only encrypting data at one place is even weaker, with the weakest being when no encryption is applied on data. To compare the selections made by participants on data encryption, we introduced a concept named protection level (PL) as a quantifiable measure. If they chose to encrypt data at one place only, the PL was 1; if they applied encryption on data at two locations, the PL value was 2; and if they chose to encrypt the data at all three locations, the PL value was 3.

On the log-in credential page, there are 4 options for log-in credentials: (1) users are required to first provide a username and the corresponding password and then a randomly generated personal identification number (PIN) for 2-factor authentication, (2) users are required to enter their username and password only, (3) users only need to enter a PIN, and (4) users are not required to enter anything to log into the system as the log-in credentials are stored on the local device. Obviously, the security strength decreases as the number of items in the log-in credentials needed for accessing the system decreases. Among these log-in options, the strongest is the 2-factor authentication (username, password, and PIN); however, this choice also requires the largest number of steps, which makes it the least convenient or with the lowest usability from the user’s perspective.

On the RBAC page, there are 2 options for RBAC: (1) not using RBAC and (2) using RBAC. Clearly, option 2 is stronger than option 1. Similarly, on the image encryption page, there are also 2 options for image encryption: (1) not using imaging encryption and (2) using image encryption. Again, option 2 is stronger than option 1.

When we designed the SecSim app, we intentionally did not design an elaborate user interface but instead used a very clear and simple design to explain the meaning of each security feature and the consequences of each option for each security feature. After all, the purpose of this app is to provide a brief security education before the user makes a decision in their security settings. An interface with a lot of details and fancy colors may actually distract users from the core content of the security education.

### Study Participant Recruitment

The participants were recruited through flyers distributed at public places in the Greater Pittsburgh area and on the Pitt+Me website at the University of Pittsburgh. Participants were screened using the following selection criteria: native English speaker, high school or higher education, aged between 18 and 65 years, capable of communicating with others verbally and in writing, and having at least a few years of experience using smart devices such as a smartphone, tablet, or smart watch.

### Study Procedure

Before the study, all study participants were required to read a consent form and sign the consent form if they were willing to participate in the study. The study participation was completely voluntary, and the participant could leave the study at any time. At the beginning of the study, a general introduction to the purpose of the study and the mobile app (SecSim) was provided, along with a brief demo of the app. All the participants were then asked to use the SecSim mobile app on an Android tablet (Samsung Galaxy Tab 4 10.1 inches, 16 GB, white tablet, Android version 4.4.2) to finish the tasks described earlier, such as registering an account, making selections in security settings, and receiving the brief security education. At the end of the study, the study participants were asked to provide responses to a usability questionnaire (IBM Post-Study System Usability Questionnaire [PSSUQ]) [19] and answer a few open-ended interview questions. On the PSSUQ, study participants were asked to respond to the 19 statements, with a scale ranging from 1, meaning strongly agree, to 7, meaning strongly disagree. The study participants were asked to fill out the usability questionnaire via the Web-based Qualtrics system. The open-ended questions were used to obtain study participants’ comments and suggestions on this study, the SecSim mobile app itself, the implemented security features on the mobile app, their ideas about information security in general, and their source of security knowledge (eg, classes, friends and family) if any.

Study participants’ demographics and their responses to the PSSUQ usability questionnaire were exported into a SPSS data file. The study participants’ selections in the security settings (both before and after the security education) were also downloaded from the SecSim app. IBM SPSS version 24 was used to perform the data analysis. Mean and SD were reported for the usability study. One-way analysis of variance (ANOVA) was used to find the setting differences among the various demographic groups.

The selections made by the study participants before and after the security feature demonstration (the brief security education) were compared and assigned to 3 categories: weaker, no change, and stronger. The number of study participants in each category for each security feature was calculated. One-way ANOVA was also used to determine the setting change behavior among the various demographic groups.

## Results

### Demographics

The study was conducted from May 2017 to September 2017 in the Greater Pittsburgh area. In total, 66 participants were recruited to undertake the study. The mean age of participants was 31.1 years (SD=13.42). More specifically, there were 40 participants (40/66, 61%) aged 18 to 28 years, 16 participants (16/66, 24%) aged 29 to 50 years, and 10 participants (10/66, 15%) aged 51 to 65 years. The gender of participants was balanced. There were 31 males (31/66, 47%) and 35 females (35/66, 53%). There were 11 African Americans (11/66, 17%), 38 white Americans (38/66, 58%), and 17 Asian Americans (17/66, 26%). Furthermore, 25 participants (25/66, 38%) had received an associate’s degree or lower education, 17 (17/66, 26%) had a Bachelor’s degree, and 24 (24/66, 36%) had a graduate degree. A total of 51 (51/66, 77%) participants were single, 13 (13/66, 20%) were married or in a long-term committed relationship, and 2 (2/66, 3%) were divorced or separated. Overall, 48 (48/66, 73%) participants lived in an urban area, 16 (16/66, 24%) lived in a suburban area, and 2 (2/66, 3%) lived in a rural area. Most of these study participants (45/66, 68%) had a part-time or full-time job, 17 (17/66, 26%) were not employed, and the other 4 participants (4/66, 6%) were retired or disabled. These participants had diverse occupations, including student, researcher, administrative personnel, and customer services personnel such as chef, bartender, other restaurant service person, as well as teacher, professor, attorney, and census field representative.

The participants were asked to perform a self-assessment on their own health status, rating it as excellent, very good, good, fair, or poor. None chose poor. In total, 3 (5%) participants chose fair, 19 (19/66, 29%) selected good, 24 chose very good (24/66, 36%), and the rest (20/66, 30%) claimed their health was excellent. Overall, 19 (19/66, 29%) of these participants used Android-based smartphones or tablets, 44 of them (44/66, 67%) used iOS-based mobile devices, and the other 3 participants used different mobile operating systems. The average number of years of experience using smart devices was 6.0 (SD=2.59). More than half of these participants (38/66, 58%) had used mHealth apps such as Apple Health, MyFitnessPal, MyChart, Fitbit app, Pink Pad, Clue, SnoreLab, 10% Happier, 7 Minute Workout, Garmin Connect, and Samsung Health. The household income of the study participants fit into 6 categories: less than US $10,000 (13/66, 20%), between US $10,001 and US $25,000 (14/66, 21%), between US $25,000 and US $50,000 (18/66, 27%), between US $50,000 and US $100,000 (7/66, 11%), greater than US $100,000 (9/66, 14%), and decline to answer (5/66, 8%). The demographic information is summarized in Table 1.

### Security Settings Before Education

As there was no simulation on password update frequency or data storage and backup locations, study participants were simply asked to make selections for the given options. As it turned out, 11 participants (11/66, 17%) chose to update their password once a month, 15 participants (15/66, 23%) chose once every 3 months, 19 participants (19/66, 29%) chose once every 6 months, 6 participants (6/66, 9%) chose once a year, 13 participants (13/66, 20%) chose never, and the remaining 2 participants (2/66, 3%) did not make any selection for this question. Furthermore, 34 participants (34/66, 52%) chose local device only for data storage and backup, 5 participants (5/66, 8%) chose remote server only, and 25 participants (25/66, 38%) chose to use both local device and remote server for data storage and backup. There were 2 participants (2/66, 3%) who did not indicate a preference on data storage and backup.

**Table 1:** Demographic characteristics of the study participants (N=66).

Demographic characteristic		Value
Age in years, mean (SD)		31.1 (13.42)
Years of using smart mobile devices (1-10), mean (SD)		6.0 (2.59)
**Age in years, n (%)**		
	18-28	40 (61)
	29-50	16 (24)
	51-65	10 (15)
**Gender, n (%)**		
	Male	31 (47)
	Female	35 (53)
**Race, n (%)**		
	Black	11 (17)
	White	38 (58)
	Asian	17 (26)
**Education, n (%)**		
	High school or lower	2 (3)
	Some college, no Bachelor’s degree	23 (24)
	Bachelor’s	17 (26)
	Graduate	24 (36)
**Marital status, n (%)**		
	Single	51 (77)
	Married or in a long-term committed relationship	13 (20)
	Divorced or separated	2 (3)
**Living place, n (%)**		
	Urban	48 (73)
	Suburban	16 (24)
	Rural	2 (3)
**Employment, n (%)**		
	Employed, working 1 to 20 hours per week	14 (21)
	Employed, working 21 to 40 hours per week	22 (33)
	Employed, working more than 40 hours per week	9 (14)
	Not employed, looking for a job	9 (14)
	Not employed, not looking for a job	8 (12)
	Retired or disabled	4 (6)
**Occupation, n (%)**		
	Student	24 (36)
	Researcher	10 (15)
	Administrative personnel	6 (9)
	Customer service	5 (8)
	Retired, disabled, unemployed	4 (6)
	Other	14 (21)
	No answer	3 (5)
**Self-assessed health status, n (%)**		
	Excellent	20 (30)
	Very good	24 (36)
	Good	19 (29)
	Fair	3 (5)
**Mobile OS, n (%)**		
	Android	19 (29)
	iOS	44 (67)
	Other	3 (5)
**Used mobile health apps?, n (%)**		
	Yes	38 (58)
	No	28 (42)
**Household income, n (%)**		
	≤US $10,000	13 (20)
	US $10,001–US $25,000	14 (21)
	US $25,001–US $50,000	18 (27)
	US $50,001–US $100,000	7 (11)
	>US $100,000	9 (14)
	Decline to answer	5 (8)

Table 2 lists the number and percentage of study participants who chose the specific options in those security features before and after they received the security training in the SecSim app. One-way ANOVA was used to determine whether their initial security settings were significantly different for participants having different demographic characteristics. The results indicated that responses to security settings did not differ significantly (*P*>.05) for participants of different ages, gender, race, marital status, living environment, employment status, household income, mobile operating system, occupation, or education.

However, password update frequency did differ significantly (*F*_3,60_=5.208, *P*=.003) for participants in different self-assessed health status categories. More specifically, participants who reported being in *very good* health did not want to change their password as frequently as those who reported being in *good* health. One possible reason is that participants with *very good* health believed that they did not have much highly sensitive health information to protect. This was confirmed by the responses from participants who claimed to have *excellent* health, which are similar to the ones from participants with *very good* health (*P*=.98). Therefore, the difference in the password update frequency between participants with *excellent* and *good* health was also large, although it was not statistically significant (*P*=.08). Here, we ignored the responses from participants with *fair* health because of the small number of participants in that category (n<5). This rule (not including categories with less than 5 participants) applies for all of the other one-way ANOVA analysis results below as well.

### Security Setting Changes After Education

All the study participants were able to finish all the assigned tasks easily in approximately 10 min. None of them had any significant difficulties using the mobile app during the study. After the brief security education, study participants performed another round of security preference selection (see Table 2, last column). These selections made in this round were compared with the initial selections made before the security education, and the results were arranged into 3 categories: stronger, weaker, and no change, as shown in Table 3.

In terms of encryption, 21 participants (21/66, 32%) chose to use a stronger measure after education; for instance, instead of only encrypting the data on the local device or remote server, they chose to encrypt data at both locations or at all 3 places. No one chose to use a weaker security measure after the security education.

There were 33 (33/66, 50%) study participants who wanted to use RBAC before the security education; after the education, that number increased to 53 (53/66, 80%), that is, 20 (20/66, 30%) participants chose to use a stronger security measure in access control. Only 1 (1.5%) participant chose to use a weaker security measure.

There were 44 participants (44/66, 67%) who wanted to use image encryption in the initial selection; after the security education, 58 participants (58/66, 88%) wanted to use image encryption. In other words, 14 (14/66, 21%) more participants chose to use a stronger security measure for image protection. Only 1 participant (1/66, 2%) chose a weaker security measure for image protection.

**Table 2:** Six security features implemented in the SecSim app, their options, and the selections made by 66 study participants before and after the security education.

Feature label		Feature description	Before, n (%)	After, n (%)
**Encryption**				
	1	Encrypting data on local device (PL^a^=1)	28 (42)	18 (27)
	2	Encrypting data when transmission (PL=1)	6 (9)	5 (8)
	3	Encrypting data on the remote server (PL=1)	13 (20)	7 (11)
	1,2	Encrypting data on local device and during transmission (PL=2)	0 (0)	2 (3)
	1,3	Encrypting data on local device and remote server (PL=2)	1 (2)	0 (0)
	2,3	Encrypting data during transmission and on remote server (PL=2)	3 (5)	5 (8)
	1,2,3	Encrypting data on local, remote device and during transmission (PL=3)	14 (21)	29 (44)
		No answer	1 (2)	0 (0)
**Password update frequency**				
	1	Once a month	11 (17)	—^b^
	2	Once every 3 months	15 (23)	—
	3	Once every 6 months	19 (29)	—
	4	Once a year	6 (9)	—
	5	Never	13 (20)	—
		No answer	2 (3)	—
**Data storage and backup location**				
	1	On local device only	34 (52)	—
	2	On remote server only	5 (8)	—
	3	Both on local device and remote server	25 (38)	—
		No answer	2 (3)	—
**Log-in credential**				
	1	Username, password, and PIN are required	5 (8)	10 (15)
	2	Username and password are required	24 (36)	18 (27)
	3	Only PIN is required	16 (24)	16 (24)
	4	Nothing is required, all saved on the local device	20 (30)	21 (32)
		No answer	1 (2)	1 (2)
**RBAC^c^**				
	1	Not using RBAC	31 (47)	13 (20)
	2	Using RBAC	33 (50)	53 (80)
		No answer	2 (3)	0 (0)
**Image encryption**				
	1	Not using image encryption	20 (30)	8 (12)
	2	Using image encryption	44 (67)	58 (88)
		No answer	2 (3)	0 (0)

^a^PL: protection level.

^b^Not applicable.

^c^RBAC: role-based access control.

**Table 3:** A summary of the changes in security option selection after security education (N=66).

Security features	Stronger, n (%)	Weaker, n (%)	No change, n (%)
Encryption (local, remote, and transmission)	21 (32)	0 (0)	45 (68)
Log-in credentials	8 (12)	7 (11)	51 (77)
Role-based access control	20 (30)	1 (2)	45 (68)
Image encryption	14 (21)	1 (2)	51 (77)

In these 3 categories (encryption, RBAC, and image encryption), the number of participants who chose a stronger security measure is much larger than the ones who chose a weaker security measure after the education.

However, the change for the log-in setting was quite different. Only 8 participants (8/66, 12%) chose to use a stronger measure during log-in, whereas 7 participants (7/66, 11%) actually chose to use a weaker measure during log-in. The vast majority of them (51/66, 77%) chose not to change their original selection. This is expected as the participants *experienced* all 4 different log-in procedures, which required different number of steps in the app, and they wanted to balance convenience and security. Their reasoning was also confirmed by the answers to the brief interview questions at the end of the study (described in a later section).

One-way ANOVA was used to determine whether the changes in settings were significantly associated with any demographic characteristics. Participants in different age, gender, race, living place, mobile operating system, occupation, and education groups did not show a statistically significant difference in their security setting behavior after the security education. On the other hand, people in different marital status, years of experience using mobile devices, household income, employment status, and health status groups showed significantly different security setting behavior after the education.

#### Marital Status

Participants in different marital status groups had different setting behavior for image encryption (*F*_2,63_=3.373, *P*=.04). For the single participants, only 14% (7/51) switched to a stronger protection (using image encryption) and 2% (1/51) switched to a weaker security (not using image encryption), whereas almost half of the married participants 46% (6/13) switched to a stronger security and none of them switched to a weaker security after the security education.

#### Years of Using Mobile Devices

Participants having different amounts of experience of using mobile devices showed a statistically significant difference in choosing options for image encryption (*F*_2,63_=3.870, *P*=.03). More specifically, for the participants with 3 to 5 years’ experience using mobile devices, only 13% (3/24) changed to a stronger security measure and 4% (1/24) changed to a weaker security measure. For the participants with more than 5 years of experience using mobile devices, 35% (12/34) switched to a stronger security protection and none of them switched to a weaker security.

#### Income

Participants in different income groups showed a statistically significant difference in setting change behavior for RBAC (*F*_5,60_=3.846, *P*=.004). For the participants with income between US $10,001 and US $25,000, only 7% (1/14) switched to a stronger protection (using RBAC) and 7% (1/14) switched to a weaker security (not using RBAC). For the participants with an income greater than US $100,000, 78% (7/9) changed to a stronger security measure and none of them switched to a weaker security measure after the security education.

#### Employment and Health Status

Participants in different employment groups had different setting change behavior for encryption (*F*_5,60_=2.807, *P*=.02); however, none of the compared pairs of groups showed a statistically significant difference. Similarly, participants in different health status groups had different setting change behavior for log-in credential selection (*F*_3,62_=2.816, *P*=.046); however, none of those compared pairs of groups had a statistically significant difference.

### Usability Study Results

As mentioned above, the PSSUQ usability questionnaire contained 19 statements for which study participants were required to choose answers on a scale of 1 to 7, where 1 meant strongly agree and 7 meant strongly disagree. Table 4 shows the average and SD of the 66 study participants’ responses to each statement.

It is clear that most of the average values were around 2 out of 7; in other words, these study participants agreed with almost all the statements, indicating good usability. The exception was the score for statement 9: *the system gave error messages that clearly told me how to fix the problems*. In most cases, if the study participants paid attention during the demo session at the beginning of the study and strictly followed the instructions given by the investigator, the app would not generate any error messages as everything they did was correct. Only if the study participant did not follow the instructions or did not enter the correct information at the right place, would the error message pop up. Therefore, a large portion of these study participants finished the entire study without any problem and did not see any error message, and therefore, they were not sure how to respond to the statement about error message. This issue is quite common in many other usability studies using PSSUQ, and a higher value in this statement does not indicate a poor usability [19].

**Table 4:** A summary of usability study results.

Post-Study System Usability Questionnaire	Mean (SD)
1. Overall, I am satisfied with how easy it is to use this system	1.86 (0.892)
2. It was simple to use this system	1.97 (1.067)
3. I could effectively complete the tasks and scenarios using this system	1.95 (1.101)
4. I was able to complete the tasks and scenarios quickly using this system	1.97 (1.109)
5. I was able to efficiently complete the tasks and scenarios using the system	1.95 (1.044)
6. I felt comfortable using this system	2.03 (1.136)
7. It was easy to learn to use this system	1.89 (1.125)
8. I believe I could become productive quickly using this system	2.02 (1.130)
9. The system gave error messages that clearly told me how to fix the problems	3.18 (1.300)
10. Whenever I made a mistake using the system, I could recover easily and quickly	2.47 (1.205)
11. The information (such as on-line help, on-screen messages and other documentation) provided with this system was clear	2.30 (1.277)
12. It was easy to find the information I needed	2.26 (1.256)
13. The information provided for the system was easy to understand	2.23 (1.225)
14. The information was effective in helping me complete the tasks and scenarios	2.08 (1.042)
15. The organization of information on the system screens was clear	2.15 (1.218)
16. The interface of this system was pleasant	2.85 (1.765)
17. I liked using the interface of this system	2.68 (1.561)
18. This system has all the functions and capabilities I expect it to have	2.11 (1.083)
19. Overall, I am satisfied with this system	2.15 (1.167)

### Interview Results

At the end of the study, the study participants were asked a few open-ended questions to elicit comments on and suggestions for this study, the mobile app itself, security features on mobile apps, their ideas about information security in general, and their source of security knowledge. Their answers are summarized briefly below.

All the study participants welcomed this type of study and expressed an interest in knowing more about security features. They believed the app was very easy to use and the security simulations were easy to understand. Many study participants mentioned that they wished to have a more colorful and graphical user interface. Some older study participants also mentioned the font size, expressing a desire to be able to adjust the font size to meet their needs. Below are some specific comments from the study participants. Please note, as study participants were randomly selected among 238 candidates, the study participants’ IDs were numbered 1 to 238:

I like that I can see the consequence of those options visually in the encryption part.

Participant #66

Security education is very useful. If I can download the app I will use it again.

Participant #162

Security education, especially the role-based access control is new to me. The app can be even better if it is fancier on interface.

Participant #36

I hope to see more apps like this. It makes me more confident when I am asked to make selections.

Participant #225

The size of the texts is small. I want to be able to control the size of the texts. Button size can be larger as well.

Participant #38

All participants said that they knew about the basic security features offered by smart devices, such as a passcode to access a locked screen, before the study. They reported that they expected mHealth apps to protect their PHI, but at the same time, they did not like to enter log-in credentials every time, and only a small number of them were willing to use strong authentication methods. They liked to see the difference between encrypted and not encrypted data, the RBAC, and the outcome of image encryption. The latter two were new for most study participants.

All these study participants knew that information security was a big challenge, and they hoped mobile app developers could offer strong but convenient security protection to protect their PHI. The frequently mentioned sources of security knowledge were family members and friends. All these study participants had discussed information security issues with their family members or friends at different levels. Some were brief chats because of a recent news report, and some were in-depth conversations. Only a few study participants reported having taken security-related classes before this study.

## Discussion

### Principal Findings

The goal of this project is to demonstrate several commonly used security features, such as encryption, user authentication, and access control, in a mobile app and to determine whether this brief and informal security education is effective in encouraging users to choose stronger security measures to protect their personal health data.

In this study, a number of demographic characteristics were collected; however, almost none of these demographic characteristics made a significant difference on the initial security settings before the security education. In other words, the general population’s understanding of information security and their preferences were highly similar before they had security education. Results did show that health status may have an impact on the selection of password update frequency. This could be because people with excellent or very good health status may not have much health information to protect and, therefore, may not feel it is necessary for them to update the password frequently.

Although it is known that providing security education can be useful for mHealth users to choose a stronger security approach to protecting personal health data, it is not feasible to give lectures and classes to every mHealth app user. Therefore, the challenge is to determine an effective and also cost-efficient approach to provide the desired security education. In this study, instead of providing formal security tutorials, we implemented those commonly used security features into the SecSim app and guided the app users to *experience* the difference after they choose different security options. This is the so-called *learning by doing* education approach (“For the things we have to learn before we can do them, we learn by doing them.” by Aristotle, The Nicomachean Ethics).

After this brief and informal security education, participants showed significant changes in settings for encryption, RBAC, and image encryption. A significant percentage of study participants chose to use a stronger security measure after the education. In certain cases, demographic characteristics contributed to these changes; for instance, participants with different marital status or years of experience using mobile devices made statistically significant different decisions in using image encryption. A significantly larger percentage of married participants chose to use image encryption after the security education. Participants with more years of experience using mobile devices also tended to use image encryption. Similarly, a larger percentage of people with higher income (>US $100,000) chose to use RBAC. Participants in different employment groups and with different health status also showed statistically significant difference in setting change behavior on encryption after the security education. In other words, this brief and informal security education was effective in encouraging users to choose stronger security protection, which, in turn, may help them to better protect their personal health data, including PHI.

On the other hand, the security education did not produce a similar significant level of change in the log-in credential setting. The difference is that the other security features (eg, encryption and RBAC) are handled by the information system itself; therefore, these features may only have a slight impact on the performance of the information system and do not require the user to do any extra work. However, different selection on the log-in credentials’ requirement can dramatically impact a user’s experience in the information system. For instance, if they choose to use the 2-factor authentication (username, password, and a PIN), they need to first enter the username and password and manually retrieve a PIN before they can log into the system; however, if everything in the log-in credentials is stored in the system, all they need to do is to click on the icon of the app or URL of a Web portal and they immediately have access to the content of the system. In other words, although the log-in simulation can show them the differences in terms of security when using different authentication approaches, that knowledge may not be able to change their behavior as they need to have a balance between information security and *usability* of a system. If an app is accessed frequently, it becomes tedious and even annoying if complex user authentication is required for each access [20]. Therefore, although mobile app developers may be able to offer highly secure solutions in their apps, users may be reluctant to use the security features or the apps if they have poor usability [15]. Hence, mobile app developers need to use creative approaches to implement highly secure and also highly user-friendly apps.

One may notice that a large percentage of study participants did not make changes to their security settings after the security education (see Table 3). This is not necessarily a bad thing as some study participants had already obtained security knowledge from other sources (such as family members, friends, job training, and classes) as indicated in the summary of the brief interview and, therefore, chose a strong security protection from the beginning (eg, 67% participants wanted image encryption and 50% participants wanted RBAC before the security education). After the security education, 88% participants chose to use image encryption and 80% participants chose to use RBAC (see Table 2). In other words, after the security education, only a small percentage of study participants still wanted to use weaker security protection. For these people, further work is needed, such as a different type of security education.

The results of this study indicate that even a brief and informal security education as shown in this study can be effective and cost-efficient in providing the desired education to mobile app users. The SecSim app itself is small and may be embedded into other mobile apps. Before the app users are required to make decision on their security settings, they can choose to go through the security training offered by SecSim, which takes approximately 10 min. This brief and informal security training may encourage the app users to choose stronger security measures, even if sometimes the app users may have to sacrifice some convenience. If a large number of mHealth apps choose to adopt this embedded training approach (not necessarily using this SecSim app), the number of PHI breaches because of users’ (patients and health care providers) reason could be reduced. For instance, if the PHI on a mobile device is encrypted with a strong encryption algorithm such as Advanced Encryption Standard, even if the mobile device is lost or stolen, the PHI is still protected by the encryption and a data breach will not occur.

### Comparison With Prior Studies

As mentioned earlier in the Introduction section, mHealth app users have security and privacy concerns when they use mHealth apps to access or manage their personal health data [5,8,11,21]. However, many of them do not have a clear understanding of the security features offered by mobile operating systems and mobile apps, and therefore, many of them either do not use any security protection methods or do not know which settings are stronger [14,22,23]. As improving security awareness among IT system end users is considered the most cost-effective security control, a number of methods have been created to facilitate such security awareness [25]. However, all these methods, such as posters; newsletter; lectures; Web-based training; and game-based, video-based, and simulation-based training, have mainly been designed for employees in an organization, for instance, health care providers in the health care domain, not the everyday users in the general population [26].

These in-job security training are not sufficient as most mobile app users do not have access to them. The security education described in this study is more generic, and it can be used by both patients and health care providers. This study described the use of an informal, brief, but effective security and privacy training by general users. In terms of the information delivery method, the approach described in this study can be categorized into simulation-based training [26,27].

### Limitations

In this study, a large percentage of the study participants were young and highly educated (Bachelor’s degree or higher). However, our results showed that the level of education and age did not significantly affect participants’ initial selection of security options or their behavior after the brief security education. In other words, a higher education level or younger age does not necessarily mean better understanding of information security. Therefore, the study results may not change dramatically if it is done in study participants with lower education levels or older ages.

In our sample, there were a small number of people with *fair* health condition and no participants with *poor* health. Therefore, the study results may not be applicable to people with poor health conditions. According to the results from other studies, patients with severe diseases, such as heart failure and kidney transplant, do not pay much attention to their privacy but instead pay attention to receiving health care services they need [28,29].

In this study, there were 2 study participants (2/66, 3%) from the rural area. Therefore, the conclusion may not be applicable to people in the rural area. To make conclusions about the populations in the rural areas, a study with more participants from the rural areas is needed.

Although the number of participants in different household income categories was sufficient for the ANOVA analysis, the number of participants in the category with high household income (>US $100,000) was relatively small (n=9). Since the behavior of participants was dramatically different between the 2 income groups (7% switched to stronger protection among 14 participants with incomes between US $10,001 and US $25,000 vs 78% switched to stronger protection among 9 participants with income greater than US $100,000), we do not believe a bigger sample will change the conclusion. On the other hand, a bigger sample would surely make the result more convincing.

When the study participants were recruited for this study, there was no differentiation with respect to occupation. We accepted any mobile app users, whether health care providers or patients. However, as the focus of this study was personal health data protection using security features, the role of these study participants was closer to that of patients, even though the same security knowledge received by health care providers can be applied to patient data protection. A future area of study could be to conduct a similar study but making the settings closer to health care providers’ work environment and then recruiting health care providers to be participants.

### Conclusions

In this study, a brief and informal security education was delivered to the mobile app users, and their changes in behavior were observed. The results indicated that this simulation-based security education could be helpful for encouraging users to choose stronger security measures to protect their personal health information. In the future, this type of education may be integrated into websites and mobile apps for users to view before they make selections in security settings, which may eventually improve users’ information security awareness and reduce the number of PHI data breaches.

## Abbreviations

ANOVA: analysis of variance
IT: information technology
mHealth: mobile health
PHI: Protected Health Information
PIN: personal identification number
PL: protection level
PSSUQ: Post-Study System Usability Questionnaire
RBAC: role-based access control
TLS: Transport Layer Security
